# Dominant deafness–onychodystrophy syndrome caused by an *ATP6V1B2* mutation

**DOI:** 10.1002/ccr3.761

**Published:** 2017-02-08

**Authors:** Ibis Menendez, Claudia Carranza, Mariana Herrera, Nely Marroquin, Joseph Foster, Filiz Basak Cengiz, Guney Bademci, Mustafa Tekin

**Affiliations:** ^1^ John P. Hussman Institute for Human GenomicsUniversity of Miami Miller School of MedicineMiamiFloridaUSA; ^2^ Institute for Research on Genetic and Metabolic DiseasesINVEGEMGuatemala CityGuatemala; ^3^ Department of Human GeneticsDr. John T. Macdonald FoundationUniversity of Miami Miller School of MedicineMiamiFloridaUSA

**Keywords:** *ATP6V1B2*, deafness–onychodystrophy–osteodystrophy–mental retardation–seizures, dominant deafness–onychodystrophy, whole‐exome sequencing, Zimmermann–Laband syndrome

## Abstract

Our report clarifies the role of *ATP6V1B2* in patients with deafness and onycho‐osteodystrophy and confirms that a recurring *ATP6V1B2* c.1516C>T [p.(Arg506*)], variant causes dominant deafness–onychodystrophy (DDOD) syndrome.

## Introduction

Dominant deafness–onychodystrophy (DDOD; MIM 124480), deafness–onychodystrophy–osteodystrophy–mental retardation–seizures (DOORS; MIM 220500), and Zimmermann–Laband (ZL; MIM 135500) syndromes are characterized by the association of sensorineural deafness and onychodystrophy. DDOD and ZL are autosomal dominant conditions, and DOORS is autosomal recessive. Patients with DDOD syndrome have normal development and cognitive functions 1, 2, 3, while those with DOORS and ZL syndromes, intellectual disabilities and seizures have been reported 4, 5. Additional findings in ZL syndrome include gingival enlargement, hypertrichosis, joint hyperextensibility, and hepatosplenomegaly. Pathogenic variants in *TBC1D24* (MIM 613577) 4 and *KCNH1* (MIM 603305) 5 cause DOORS and ZL syndromes, respectively. A pathogenic c.1516C>T [p.(Arg506*)] variant in *ATP6V1B2* has recently been reported to cause DDOD syndrome in three simplex cases 6. In a subsequent report, however, another *ATP6V1B2* variant c.1454G>C [p.(Arg485Pro)] was reported to cause ZL syndrome in two simplex cases 5. It remains unknown whether reported patients with DDOD syndrome and an *ATP6V1B2* variant had additional findings of ZL syndrome because those details were not available in the original report 6. Here, we describe our clinical and molecular studies in the diagnosis of a simplex patient with deafness–onychodystrophy.

## Patient Report

**Figure 1:**
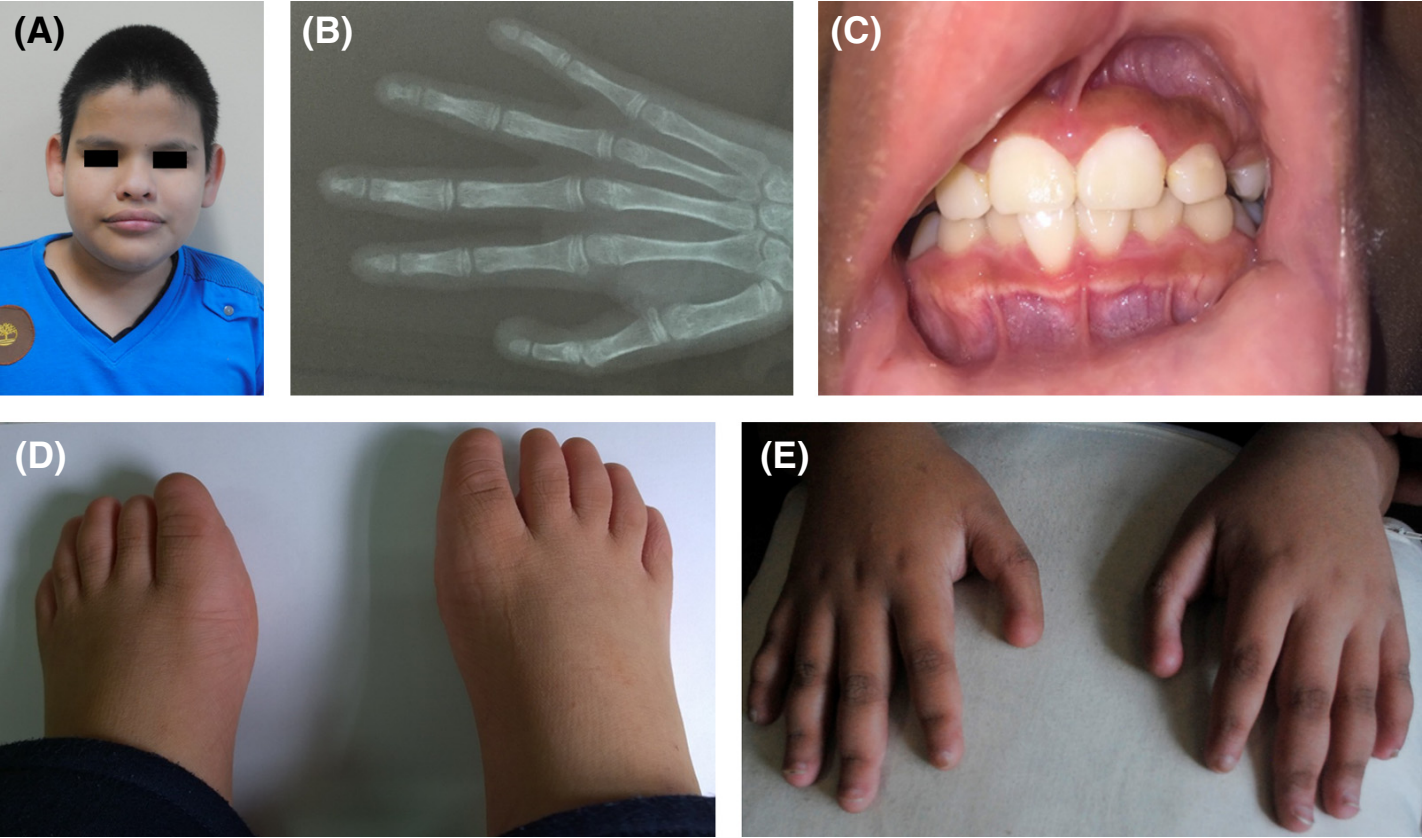
(A) Normal face, (B) X‐ray of the right hand triphalangeal thumb, (C) No gingival hyperplasia, and (D and E) aplastic/hypoplastic fingernails and absent of all toenails.

**Figure 2:**
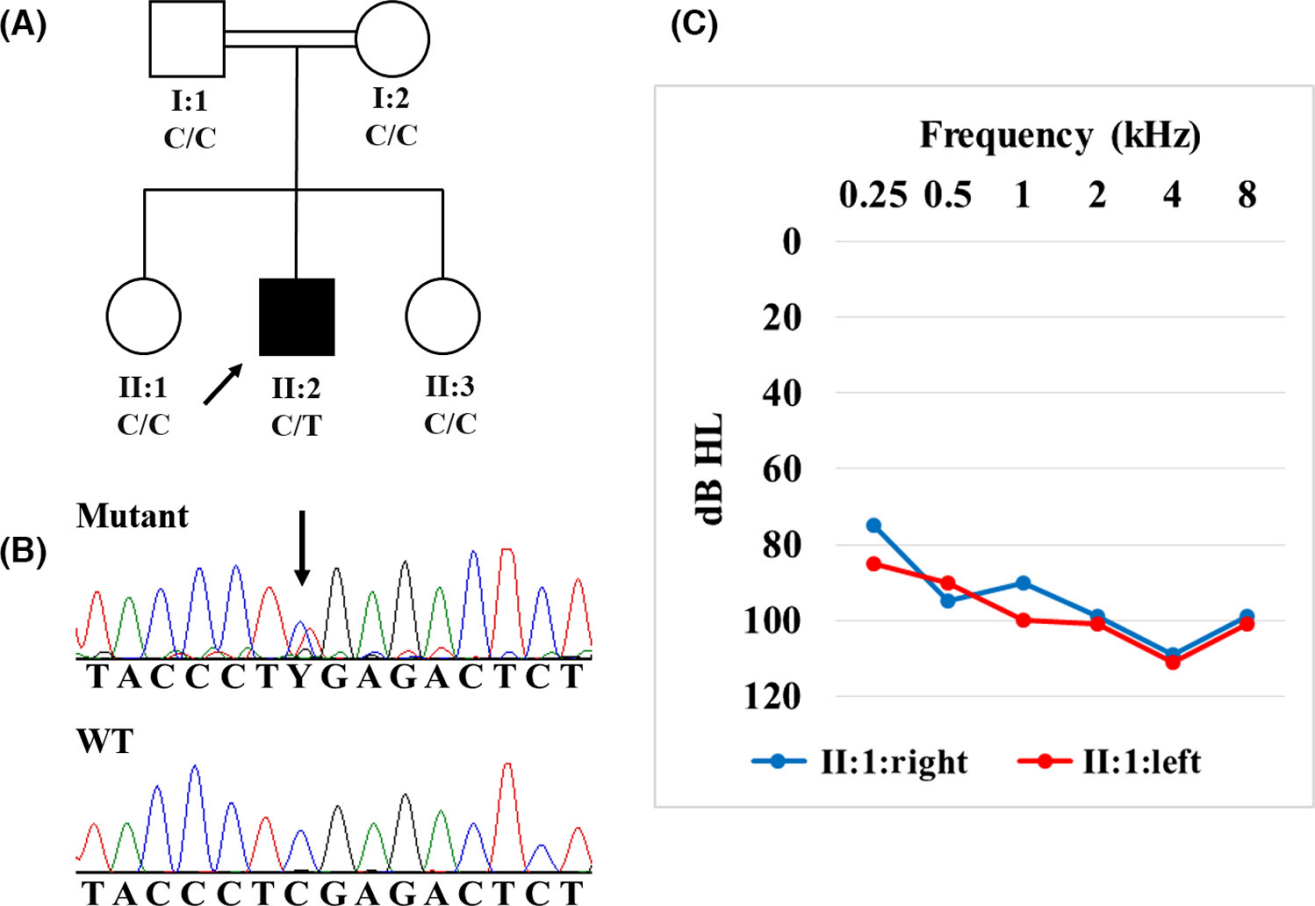
(A) Pedigree, (B) partial sequence of exon 14 in *ATP6V1B2* gene showing the heterozygous c.1516C>T [p.(Arg506*)] variant in the proband and the wild‐type sequence, and (C) audiogram showing bilateral sensorineural hearing loss.

The proband is the second child of nonconsanguineous healthy Guatemalan parents. Two sisters are healthy (Fig. 1). He was born via normal spontaneous vaginal delivery at term following an uneventful pregnancy. Birth weight was 3060 g. Initial examination revealed bilateral digital anomalies in hands and feet. Audiological examination by auditory brainstem responses (ABR) at 1 year of age indicated profound bilateral sensorineural hearing impairment. At the age 12 years, his height, weight, and head circumference measure 146 cm (25–50 percentile), 48 kg (75–90 percentile), and 56 cm (90 percentile), respectively. He has a high forehead with dolichocephaly, bilateral triphalangeal thumbs without nails, hypoplastic fingernails from second to fifth fingers, flat feet with absent toenails (Fig. 1). He does not have gingival hyperplasia, hypertrichosis, organomegaly, or joint hyperextensibility. Neurological examination followed by EEG, brain CT scan, and MRI did not show abnormalities. He does not communicate orally, but a rudimentary sign language was present. Bilateral sensorineural severe‐profound hearing loss was documented (Fig. 2). Family history was negative for deafness, nail dysplasia, and intellectual disabilities.

This study was approved by the University of Miami Institutional Review Board (USA) and Guatemala National Health Ethics Committee. After written informed consents were collected, DNA was extracted from peripheral blood samples by standard procedures. Whole‐exome sequencing in the proband was performed as previously reported 7.

Whole exome sequencing (WES) was performed only in the proband 7. Targeted exonic regions were covered 100%, 96%, and 88% in read depth of 1X, 5X, and 10X, respectively. In total, 82,594,033 number of reads, 92,131 base substitutions (synonymous, nonsynonymous, intronic), and 8277 insertions/deletions were detected. Variants were filtered as previously published 7 in all modes of inheritance patterns, followed by filtering for Mendelian genes previously reported in OMIM Morbid.

The proband was found to be heterozygous for c.1516C>T [p.(Arg506*)] in *ATP6V1B2* (NM_001693.3). Sanger sequencing confirmed the variant only in the proband and excluded the variant in parents and sisters (Fig. 2). We did not test the parental identities with additional markers.

## Discussion

Deafness and onychodystrophy, although major diagnostic findings, fail to guide specific clinical diagnosis. Seizures and intellectual disabilities are used to differentiate DOORS from DDOD 1, 4, 6, 8. Neurological and behavioral problems are common in children with severe‐profound hearing loss. For instance in our patient, severe‐profound prelingual sensorineural hearing loss and a long period of auditory deprivation without specific education led to a phenotype with limited social interactions. Time of deprivation, and nutritional and socioeconomic status have been associated with developmental delay and cognitive problems in deaf individuals 9, 10. While ZL syndrome has additional features such as gingival enlargement and hypertrichosis, differential diagnosis is not always straightforward.

Interestingly, pathogenic variants in *ATP6V1B2* have been reported to cause both DDOD and ZL syndromes 5, 6. Table 1 summarizes phenotypic findings in *ATP6V1B2‐*related disorders. It should be noted that the information about differentiating clinical features between DDOD and ZL syndromes was missing in the report associating an *ATP6V1B2* variant with DDOD syndrome 5 (Table 1). In our patient, the p.(Arg506*) variant does not cause gingival hyperplasia, hypertrichosis, or organomegaly and is associated with DDOD syndrome. Individuals with the p.(Arg485Pro) variant on the other hand were reported to develop these additional findings and are diagnosed with ZL syndrome.

**Table 1:** Genetic and clinical characteristics of individuals with deafness‐onychodystrophy syndrome and/or *ATP6V1B2* mutations

	Our patient	Yuan et al. 6	Vind‐Kezunovic et al. 2	White et al. 3	Kortüm et al. 5
No. of affected individuals	1	3	3	3	2
Diagnosis	DDOD	DDOD	DDOD	DDOD	ZLS
Gene	*ATP6V1B2*	*ATP6V1B2*	ND	ND	*ATP6V1B2*
Mutation	p.(Arg506*)	p.(Arg506*)	ND	ND	p.(Arg485Pro)
Coarse facies	–	–	–	–	2/2
Absent/hypoplastic finger nails	1/1	3/3	3/3	3/3	2/2
Deafness	1/1	3/3	3/3	3/3	1/2
Thumbs	Triphalangeal thumb	–	3/3 (Finger‐like)	1/2 (Long, Finger‐like)	1/2 (Elongated)
Absent/hypoplastic toe nails	1/1	3/3	3/3	NR	2/2
Aplastic/hypoplastic phalanges	1/1	3/3	1/3	NR	2/2
Brachydactyly	1/1	3/3	3/3	1/2	2/2
Scoliosis	–	NR	NR	NR	1/2
Gingival enlargement	–	NR	NR	NR	2/2
Hypertrichosis	–	NR	NR	NR	2/2
Intellectual disability	–	–	1/3	–	2/2
Inheritance	De novo^a^	De novo	AD	AD	De novo

–, absent; ND, not determined; NR, not reported finding; AD, autosomal dominant; ZLS, Zimmermann–Laband syndrome; DDOD, dominant deafness–onychodystrophy syndrome.

a While samples from neither parent shows the variant, parental identities were not checked with DNA markers.

## Conflict of Interest

The authors declare no conflict of interests.

## Authorship

All authors contributed extensively to the work presented in this study. IM, CC, GB, and MT: performed clinical examination, interpreted the data, and wrote the manuscript. MH, NM, JF, and FBC: draw blood samples, obtained DNA, and conducted genetic studies.
